# In vivo Assessment of the Potential Protective Effects of Traditional Kumis Against SARS-CoV-2 in a Transgenic Mouse Model

**DOI:** 10.1007/s12560-026-09692-4

**Published:** 2026-04-20

**Authors:** Bilal Kocaman, Hatice Nur Aydin, Fatih Ramazan Istanbullugil, Mitat Sahin, Irem Abaci Bayraktar, Arzu Tas Ekiz, Ozge Aksoy Yaman, Hivda Ulbegi Polat

**Affiliations:** 1 TUBITAK Marmara Research Center, Presidency of Climate and Life Sciences, 41470 Kocaeli, Türkiye; 2 Department of Biotechnology, Institute of Biotechnology, Gebze Technical University, 41400 Kocaeli, Türkiye; 3 Department of Bioengineering, Istanbul Medeniyet University, 34700 Istanbul, Türkiye; 4 Department of Food Hygiene and Technology, Faculty of Veterinary Medicine, Kyrgyz-Turkish Manas University, Bishkek, 720042 Kyrgyzstan; 5 Department of Microbiology, Faculty of Veterinary Medicine, Kafkas University, 36300 Kars, Türkiye; 6 Molecular Biology and Genetics, Institute of Sciences, Yildiz Technical University, 34220 Istanbul, Türkiye

**Keywords:** Antiviral activity, K18-hACE2 mouse model, Kumis, Mare’s milk, SARS-CoV-2

## Abstract

This study explores the antiviral potential of kumis, a traditional fermented mare’s milk beverage, against SARS-CoV-2 using K18-hACE2 transgenic mice. Kumis samples fermented in leather, wood, and plastic containers were tested to assess their immunomodulatory and antiviral effects. Mice were intranasally infected with live SARS-CoV-2 and orally administered kumis daily for five consecutive days, starting one day prior to viral challenge, and monitored for a total of 15 days. Kumis-treated mice exhibited significantly reduced weight loss at specific time points (days 12 and 15 post-infection; *p* = 0.0310). Survival was numerically higher in kumis-treated groups (Plastic 5/6, Wooden 5/6, Leather 4/6) compared to controls (3/6), but this difference did not reach statistical significance (*p* = 0.6649). Histopathological and virological analyses supported the protective effects, with the plastic-fermented sample showing the most pronounced benefit. The study also highlights the importance of fermentation environments on the microbial composition and therapeutic efficacy of kumis. Although kumis is not a direct antiviral drug, it can serve as a supportive food that enhances immunity and provides protection during viral illnesses.

**Supplementary Information:**

The online version contains supplementary material available at 10.1007/s12560-026-09692-4.

## Introduction

Severe Acute Respiratory Syndrome Coronavirus 2 (SARS-CoV-2) is a virus that emerged in December 2019 in Wuhan, Hubei province, China, infecting a large number of individuals (Zhu et al., 2020). Due to its rapid global spread, the World Health Organization (WHO) declared COVID-19 a pandemic in March 2020 (Wu et al., 2020). SARS-CoV-2 enters host cells by binding to the angiotensin-converting enzyme 2 (ACE2) receptor on the cell membrane through the receptor-binding domain (RBD) on its spike (S) protein (Bosso et al., 2020). Consequently, some drug development strategies have focused on inhibiting the expression of this receptor (e.g., using anti-hypertensive drugs) (Asai et al., 2020).

Fermented dairy products have been utilized since ancient times both as a vital source of nutrition and for their therapeutic properties. Experimental studies have demonstrated that these products possess functional attributes beneficial to human health (Afzaal et al., 2021). Fermentation of dairy products, primarily mediated by lactic acid bacteria (LAB), though yeasts and molds may also contribute, not only detoxifies the product but also alters its taste, aroma, texture, and nutritional content while improving quality, shelf life, and digestibility (Afzaal et al., 2021; Gadaga et al., 1999; Man & Xiang, 2021).

Mare’s milk, in particular, has been traditionally consumed for millennia in Central Asia and neighboring regions owing to its rich nutritional profile, including proteins, carbohydrates, lipids, vitamins, macro- and microelements, enzymes, hormones, and other essential compounds and its ease of digestion. The term ‘kumis’ is spelled similarly in several languages, including Kumiss, kuymiss, kymyz, qymyz, qimiź and kumiz (Meng et al., 2021). In Mongolia, it is observed that various different terminologies such as “chigo,” “chigee,” “airag,” and “Kumiss” are also used (Tsuchiya et al., 2020). This beverage is acidic and mildly alcoholic, produced through a fermentation process that involves both lactic and alcoholic stages, yielding lactic acid, ethyl alcohol, carbon dioxide, and various volatile aroma compounds (Kondybayev et al., 2018; Uniacke-Lowe, 2011). Kumis typically contains between 0.6% and 3% alcohol and is fermented in specialized containers such as saba made from horsehide or wooden kubi (Afzaal et al., 2021; Kondybayev et al., 2021).

Kumis is a fermented dairy product traditionally produced in Central Asia and is widely recognized for its potential health benefits. In traditional practices, local communities ferment mare’s milk by placing it into leather, wooden, or plastic containers, where the kumis production process takes place. Primarily, it is a probiotic-rich food suitable for human consumption (Istanbullugil et al., 2025). In addition, kumis contributes to the regulation of the immune system and cholesterol levels, protects the digestive system, and helps control blood pressure and blood sugar levels while supporting the synthesis of essential vitamins (Afzaal et al., 2021). In another Central Asian country, Kazakhstan, the first kumis treatment center was established in 1854 in the Samara region. It is known that the Kazakh people used kumis for the treatment of tuberculosis in ancient times. Today, there are many kumis therapy centers across Kazakhstan, where patients receive kumis treatment under medical supervision (Erdem & Gundogdu, 2018).

Clinical studies have revealed that patients with COVID-19 experience alterations in their gut microbiota, characterized by an increased proportion of pathogenic microorganisms relative to beneficial ones (Zuo et al., 2020). This imbalance imposes additional stress on the immune system, impairing the body’s ability to effectively combat the infection (Shook et al., 2020). By enhancing the population of beneficial microorganisms, kumis consumption may help restore gut flora balance, thereby alleviating immune system strain and potentially ameliorating the course of the infection (Bousquet et al., 2020). Furthermore, animal-derived fermented products contain bioactive peptides and short-chain fatty acids (SCFAs) that support human health through antioxidant, anti-radical, and anti-hypertensive effects, as well as by mitigating lactose intolerance. Among the most common bioactive peptides produced during the fermentation of dairy, meat, and oyster products are angiotensin-converting enzyme inhibitor (ACE-I) peptides (Chen et al., 2015; Rai et al., 2017). Additionally, other phenolic compounds, flavonoids, bacteriocins, and organic acids play significant roles in neutralizing viral agents (Muhialdin et al., 2021).

Probiotics have gained increasing attention for their potential contributions to human health. Since the early 2000 s, probiotic-based fermented foods have regained popularity due to their reported health benefits (Maftei et al., 2024). Probiotics, including those derived from traditionally fermented foods, have been suggested as supportive therapeutic approaches in the management of COVID-19 and other viral infections through their immunomodulatory effects (Mgbodile et al., 2023). In a study conducted by Oba et al. (2021), the effects of natto, a traditional Japanese fermented food made from soybeans, on viral infections were investigated, particularly against SARS-CoV-2 and bovine herpesvirus type 1 (BHV-1). The researchers reported that treatment with natto extract completely inhibited infection by both SARS-CoV-2 and BHV-1. Raw materials, starter cultures, temperature, light conditions, and fermentation containers are key factors that directly influence the quality and characteristics of fermented foods (Istanbullugil et al., 2025; Marco et al., 2021; Tamang et al., 2016).

In this study, we evaluated the efficacy of kumis fermented in different fermentation containers against SARS-CoV-2 in K18-hACE2 transgenic mice using a live virus challenge model. The kumis batches used in this study were the same samples that were previously subjected to metagenomic characterization in the study by Istanbullugil et al. (2025), conducted in the same laboratories. In that study, kumis produced in plastic, wooden, and leather containers was analyzed for its microbial composition, providing detailed microbiological profiling of the batches used in the present in vivo experiments. Additionally, we compared the toxic and antiviral effects of kumis produced in different fermentation containers in the mouse challenge model. Consequently, our study provides an experimental evaluation of kumis’s potential antiviral activity and explores whether fermentation container type may influence biological outcomes.

## Materials and Methods

### Sample Collection and Fermentation Conditions

Kumis samples were obtained from traditionally fermented products produced in leather, wooden, and plastic containers on the plateau of the Susamyr region in Kyrgyzstan, where traditional production methods are still practiced. Initially, samples were collected from nine different household producers located in the same settlement; three producers used leather containers, three used wooden containers, and three used plastic containers (Istanbullugil et al., 2025). However, for the in vivo antiviral activity study, only one representative sample from each container type was selected and used. The kumis samples were collected after one day of fermentation. The mares were all raised under identical environmental conditions and grazed on the same pastures; the spontaneous fermentation process was influenced solely by the type of container used. The spontaneous fermentation of the kumis samples was carried out under ambient environmental conditions reflective of traditional practices. Three samples of each material were placed in clean, sterilized 50 mL falcon tubes, transported in an icebox, and subsequently stored at − 80 °C.

### Ethics Statement and Mice

All experimental procedures with animals were approved by TUBITAK MRC, Life Sciences, Medical Biotechnology Unit in Gebze, Turkey. All procedures in this study involving animals were reviewed and approved by the Institutional Biosafety Committee and Institutional Animal Care and Use Committee (HADYEK-16563500-111−10029); all the experiments were conducted in compliance with all relevant ethical regulations. The experiments were conducted in Biosafety Level 3 (BSL3) and Animal Biosafety Level 3 (ABSL3) facilities at TUBITAK MRC Life Sciences.

The K18-hACE2 [B6.Cg-Tg(K18-hACE2)2Prlmn/J] transgenic mice used in this study were provided by the Jackson Laboratory in the United States. TUBITAK MRC Life Sciences Experimental Animals Unit had rights for the production of K18-hACE2 transgenic mice. All of the experiments were conducted in a biocontainment isocage, which was part of ABSL 3. 8–10-week-old female K18-hACE2 transgenic mice were used, and six mice were used for each group. In this study, there were four groups based on the type of container used for storing kumis: Kumis samples stored in plastic, wooden, and leather containers, and a placebo group (PBS).

In this study, animals were randomly assigned to experimental groups prior to treatment initiation. Age and body weight were matched across groups to ensure baseline comparability. For post-mortem analyses, including RT-PCR and histopathological evaluation, samples were coded using group and animal numbers only (e.g., Group 2 – Animal 3), and investigators performing these analyses were blinded to group allocation. Body weight monitoring, survival assessment, and gross pathological evaluation were conducted by the experimental team aware of group assignments, as these parameters were recorded during the live challenge phase.

### Determination of Kumis Dosage

An acute oral toxicity assessment was performed to evaluate whether kumis samples fermented in different containers exerted any harmful effects in mice. The administered volume (200 µL/mouse) was selected based on standard oral gavage volume recommendations for mice (≤10 mL/kg body weight). Three experimental groups were established according to fermentation container type (plastic, wooden, and leather), with four K18-hACE2 transgenic mice per group. For the acute oral toxicity evaluation, a single oral gavage dose of 200 µL per mouse was administered. Animals were monitored for seven days following administration. All mice survived throughout the observation period, and no significant changes in body weight were recorded. Gross pathological examination revealed no visible abnormalities in the organs upon macroscopic inspection.

### Intranasal Delivery Method for SARS-CoV-2 Infection in Mouse Models

Since kumis is a naturally consumable daily food, it was considered a dietary supplement in this experimental study. To evaluate its effect on the immune system and assess its potential antiviral activity, a challenge study was conducted using live SARS-CoV-2 virus in mice. One kumis sample was randomly selected from each storage type among the samples received from Kyrgyzstan for administration to the experimental groups. Throughout the application period, all samples were stored at + 4 °C. Prior to administration, the samples were observed to be similar in terms of odor, color, and visual fluid appearance, regardless of the type of storage container. For the challenge experiment, transgenic mice were transferred to the BSL-3 facility. The experimental timeline was structured as follows. Kumis administration was initiated one day before viral challenge (Day − 1), when mice received 200 µL/mouse orally by gavage. On Days 2, 3, and 4, mice received kumis orally in the morning, followed by intranasal infection in the afternoon under anesthesia (ketamine 100 mg/kg and xylazine 10 mg/kg) with 50 µL of the SARS-CoV-2 B.1.36 Wuhan strain (10⁵ TCID₅₀). On Day 5, mice received the final oral dose of kumis. Thus, kumis was administered for a total of five consecutive days, while viral challenge was performed over three consecutive days. Animals were monitored for clinical signs, body weight changes, and survival for a total of 15 days from the first day of infection (Polat et al. 2024; Ulbegi Polat et al. 2023a, b). The SARS-CoV-2 B.1.36 Wuhan strain used in this study was obtained by the Ministry of Health during the COVID-19 pandemic.

Following SARS-CoV-2 infection, mice in each group were monitored daily for morbidity (as assessed by body weight) and mortality. Mice that lost more than 25% of their baseline body weight were deemed to have reached the experimental endpoint and were euthanized. Pathological exams were done after the animals were sacrificed. All abdominal organs and thoracic cavity organs were inspected with the naked eye during gross pathology. Organs that are clean and have no lesions are scored as 0, organs with edema and hyperemia are scored between 0.5 and 1, and organs with different rates of pneumonia lesions are scored between 1.5 and 3. One half of each mouse lung was used for histological investigation, while the other half was used for real-time PCR to compare viral load.

### Histopathological Analysis

For histopathological examination, tissue samples were fixed in 10% buffered formalin, dehydrated through a graded series of alcohol solutions, and embedded in paraffin wax. Sections were cut into 5 μm-thick slices, stained with hematoxylin for 5 min, and counterstained with eosin. The stained sections were then dehydrated, cleared, and mounted on slides. Slides were examined using a Zeiss Axio Vert A1 microscope (Zeiss, Oberkochen, Germany) and images were captured with Zen 2.6 Pro software (Zeiss, Oberkochen, Germany). A semi-quantitative scoring method was applied to assess inflammation. The extent of inflammation was evaluated relative to the total lung tissue and scored on a scale from 0 to 3 as follows: **0** = No inflammation, **1** = Morphologically normal lung tissue with mild erythrocyte and lymphocyte infiltration around the bronchioles, **2** = Moderate erythrocyte and lymphocyte infiltration throughout the lung, **3** = Severely impaired morphology with intense erythrocyte and lymphocyte infiltration in the lung.

### Isolation of Viruses and RNA Extraction for RT-PCR

For real-time PCR analysis, half of each mouse’s lung was collected. Lung tissues were homogenized in 2 mL of PBS at 70% amplitude for 90 s using an ultrasonic homogenizer (Bandelin HD2200.2, Germany) for virus isolation. The homogenates were centrifuged at 21,500 × g for 10 min, and the supernatants were transferred into 15 mL falcon tubes.

Viral RNA was extracted using the QIAamp Viral RNA Mini Kit (Cat. No. 52906, QIAGEN, USA) according to the manufacturer’s instructions. Detection of viral RNA was performed using the One Step PrimeScript III RT-PCR Kit (Takara, Japan), along with SARS-CoV-2 nucleocapsid gene-specific primers and probes as detailed below. All reactions were performed on a CFX96 Touch instrument (Bio-Rad, Hercules, CA, USA) with the following real-time PCR conditions: 52 °C for 5 min, 95 °C for 10 s, then 44 cycles of 95 °C for 5 s and 55 °C for 30 s. The primer and probe sequences that were used for RT-PCR are CDC recommended and FDA EUA approved NC1 and NC2 sequences. The target region was the Nucleocapsid (NC) gene of SARS-CoV-2. Primer and probe sequences were N1 Forward: 5′-GAC CCC AAA ATC AGC GAA AT-3′, N1 Reverse: 5′-TCT GGT TAC TGC CAG TTG AAT CTG-3′ N1 Probe: 5′-FAM-ACC CCG CAT TAC GTT TGG TGG ACC-BHQ1-3 N2 Forward: 5′-TTA CAA ACA TTG GCC GCA AA-3′ N2 Reverse: 5′-GCG CGA CAT TCC GAA GAA-3′ N2 Probe: 5′-FAM-ACA ATT TGC CCC CAG CGC TTC AG-BHQ1-3 (Ulbegi Polat et al. 2023a).

### Statistical Analysis

Statistical analyses were performed using GraphPad Prism version 10.5.0 (GraphPad Software, USA). A two-way ANOVA was performed for body weight data, while lung PCR Ct values and gross lung pathology scores were analyzed using one-way ANOVA followed by post hoc comparisons when appropriate. Survival analysis was conducted using the Kaplan-Meier method, and survival curves were compared using the Log-rank (Mantel-Cox) test. All data are presented as mean ± standard deviation of the mean (SEM) unless otherwise stated. A two-sided p-value of less than 0.05 was considered statistically significant.

## Results

### Investigation of Kumis in a SARS-CoV-2 Challenge Model Using Mice

**Fig. 1:**
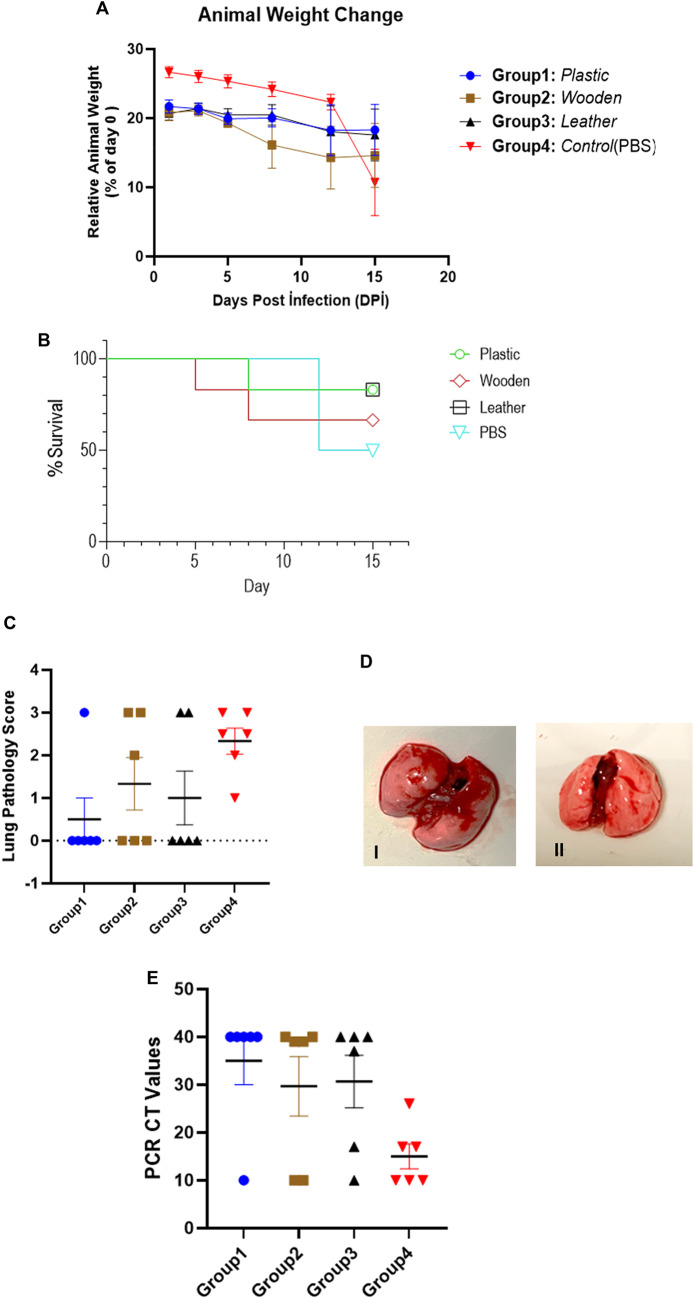
Neutralization effect of Kumis in vivo in three separate storage containers. **A** Average change in body weight over time in control and Kumis-treated mice. Body weight change differed significantly between groups over the 15-day period (*p* = 0.0310), with divergence becoming more pronounced between days 12 and 15. Statistical analysis was performed using two-way ANOVA. **B** Survival analysis was conducted using the Kaplan-Meier method. No significant difference was found between the groups (*p* = 0.6649). **C** Gross pathology inflammation scores from control and Kumis-treated mice lungs. Lung inflammation was scored from 0 to 3 (0: No inflammation, 1: Low, 2: Medium, 3: High). Statistical analysis was performed using two-way ANOVA. No significant difference was observed (*p* = 0.1275). **D** Representative lung images dissected from control and Kumis-treated mice. I: Lung infection with pneumonia (control group) II: Lung after Kumis treatment. **E** Viral loads in the lungs of control and kumis-treated mice measured by real-time PCR targeting two regions of the SARS-CoV-2 nucleocapsid gene. Statistical analysis was performed using two-way ANOVA. The result (*p* = 0.0549) indicates no significant difference in mean Ct values between groups

In the control group, clinical signs such as weight loss began to appear from 5 to 6 days post-infection (dpi) (Fig. 1A). Conversely, animals in the kumis-treated groups that showed no signs of illness did not experience significant weight loss. Between 12 and 15 dpi, mice in the control group experienced significant weight loss ranging from 22% to 33%. On day 15 dpi, three mice in the control group were found dead in the cage. In the kumis groups, one mouse each from the plastic and leather groups, and two mice from the wooden group, were also found dead on day 12 dpi. These animals had shown signs of illness and weight loss starting around day 8 dpi. Additionally, the second mouse in the leather group also began showing clinical signs and weight loss on day 8 dpi (Dinnon et al., 2020; Oladunni et al., 2020). Survival analysis of the experimental groups is presented in Fig. 1B.

Gross pathological analysis revealed pneumonic and hyperemic lesions in the lungs of the three surviving mice from the control group, whereas the lungs of the kumis-treated groups appeared clear and showed no macroscopic signs of infection. Gross pathology could not be performed on the deceased animals in the treated groups and control group, but the highest pathological scores (3) were assigned based on their clinical presentation. Additionally, hyperemic areas were observed in one mouse from the wooden group, and pneumonia was detected in the second sick mouse from the leather group (Fig. 1C). Lung gross pathology scores did not differ significantly between groups (one-way ANOVA, F (3,20) = 2.137, *p* = 0.1275). However, the group effect accounted for approximately 24% of the variance in pathology scores (R² = 0.2427), suggesting a moderate effect size. In Fig. 1D, the lungs of the control mouse that became ill during the experiment and the mouse that completed the experiment in good health were compared in terms of pneumonia.

Due to the inability to process lung tissues from all deceased animals, viral load analysis could not be performed for those samples. However, in the control group, lung RNA extracted from the three surviving mice showed high viral load, indicated by low Ct values (17 and 26). Similarly, one mouse in the leather group exhibited a low Ct value (17), indicating a high viral load. In contrast, five mice from the plastic group and four mice each from the leather and wooden groups showed no detectable viral load; for these animals, the Ct value was represented as 40, which corresponds to the maximum cycle number run by the device (Ct = 40) (Fig. 1E). Viral RNA levels did not differ significantly between groups (one-way ANOVA, F (3,20) = 2.999, *p* = 0.0549). However, the proportion of variance explained by the treatment (R² = 0.31) suggests a moderate to large effect, indicating a potential biological impact despite the lack of statistical significance. 

**Fig. 2:**
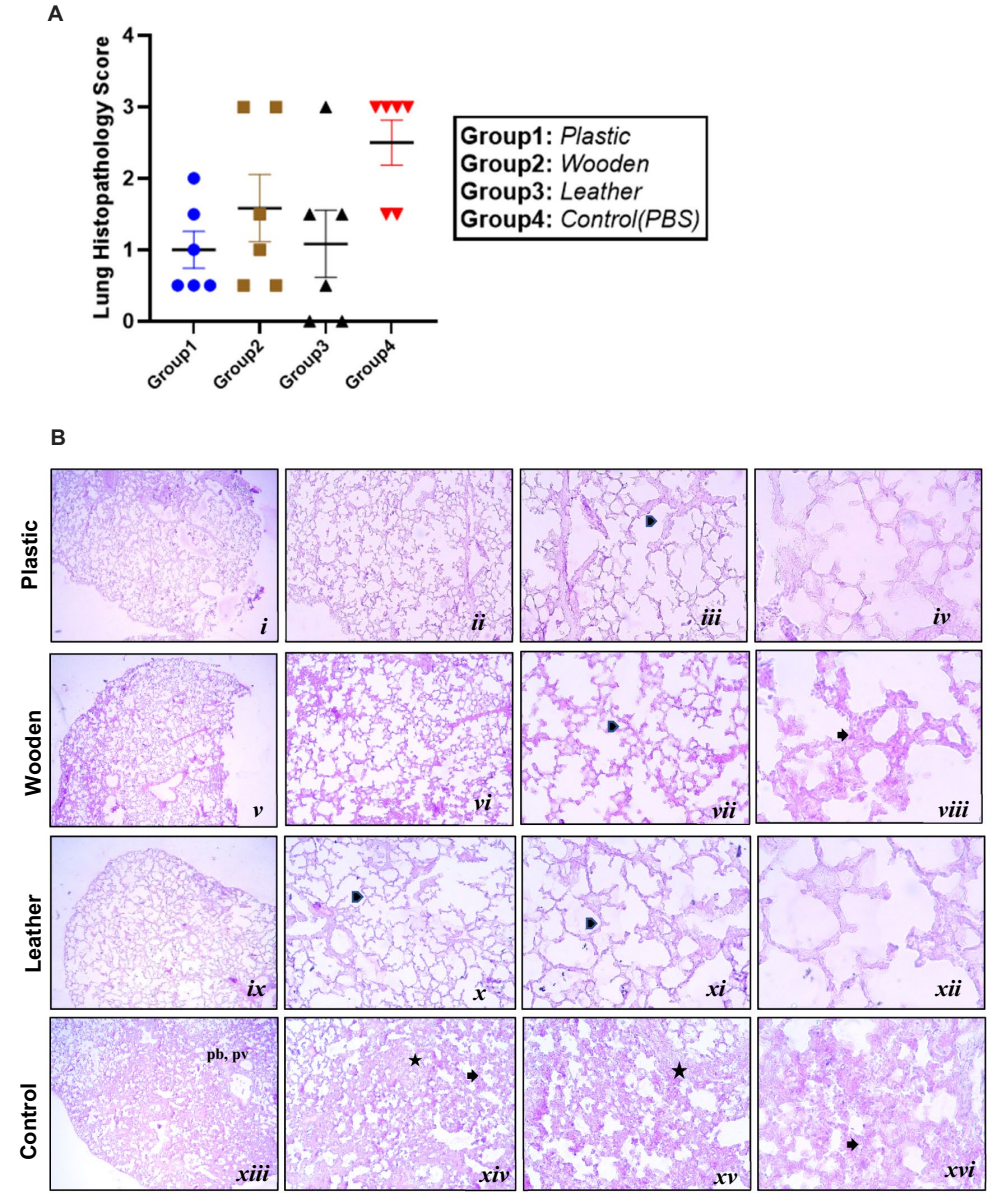
Histopathology inflammation scores from control and Kumis-treated mice lungs. **A** Lung Histopathology inflammation was scored from 0 to 3 (0: No inflammation, 1: Low, 2: Medium, 3: High). Statistical analysis was performed using two-way ANOVA. No significant difference was observed (*p* = 0.0503). **B** The IHC graph of all mice and several representative IHC images of lung tissue from the Plastic (*i-iv*), Wooden (*v-viii*), Leather (*ix-xii*) and Control (*xiii-xvi*) groups. The tissue architecture of control group shows interstitial inflammatory cell infiltration (asterisk, xiv and xv), alveolar septal thickening (arrow, xiv and xvi) and more parenchymal infiltration at peribronchiolar (pb, xiii) and perivascular (pv, xiii) regions. Wooden group shows both alveolar septal thickening (arrow, viii) and normal alveolar morphology (arrowhead, vii). Leather and plastic groups shows decreased amount of interstitial inflammatory cell infiltration and normal alveolar morphology (arrowhead, iii, x and xi). Image magnification of i, v, ix and xiii are 4x, ii, vi, x and xiv are 10x, iii, vii, xii and xv are 20x, iv, viii, xii and xvi 40x

In the treated group, histological examination of lung tissues revealed healing and restoration of normal tissue components (Kayabolen et al., 2022; Sun et al., 2020). Lung sections were analyzed after staining with hematoxylinand eosin and lung inflammation was semi-quantitatively scored between 0 and 3 (0: no inflammation, 1: low, 2: medium, 3: high). Control mice exhibited significantly higher histopathological scores compared to kumis-treated mice. The lungs of the control group displayed interstitial inflammatory cell infiltration, alveolar septal thickening, and marked parenchymal infiltration in the peribronchiolar regions. Lung pathology scores showed a borderline difference between groups (one-way ANOVA, F (3,20) = 3.092, *p* = 0.0503). Despite the lack of formal statistical significance, the treatment was associated with a measurable contribution to the variability in pathology scores (R² = 0.3168), corresponding to approximately 31.7% of the observed variance. Although kumis-treated mice demonstrated improved outcomes compared to controls, partial cellular infiltration was still evident based on gross pathology and PCR data. Figure 2A–B presents histopathological microscopy images and score graphs for the groups (Dinnon et al., 2020; Moreau et al., 2020).

## Discussion

In this study, the antiviral efficacy of kumis samples fermented in various containers using traditional methods was evaluated against SARS-CoV-2 infection in K18-hACE2 transgenic mice. The distinctive characteristics of kumis are attributed to the microbial culture used in its production, which includes LAB and yeasts (An et al., 2004). Previous studies have reported that Mare’s milk differs from cow’s milk in its higher water content and lower caloric value, with a total solids content of 10.5%, slightly lower protein (1.93%–2.1%) and fat (1.25%–1.3%) levels, and a higher lactose concentration (6.4%–6.91%) (Oftedal et al., 1983; Shaikh et al., 2022). Kumis and its probiotic content have been shown to support immune function and improve digestion in previous studies (Afzaal et al., 2021; Jastrzębska et al., 2017).

Chen et al. (2015) reported that the immune-supportive properties attributed to fermented dairy products, including kumis, may be associated with their content of probiotic bacteria, bioactive peptides, organic acids, short-chain fatty acids (SCFAs), and ACE-inhibitory peptides. These peptides have been described as capable of inhibiting angiotensin-converting enzyme (ACE), thereby potentially influencing angiotensin II synthesis and related inflammatory and oxidative pathways. ACE-inhibitory peptides identified in fermented foods have been suggested to modulate certain aspects of disease-related processes; however, such mechanisms were not evaluated in the present study (Chen et al., 2015; Muhialdin et al., 2021).

Traditional fermented dairy products are rich in bioactive substances such as peptides, amino acids, vitamins, and minerals (Shaikh et al., 2022). Kumis includes various biologically active substances, including lactoferrin, angiogenin, and vital polyunsaturated fatty acids, as well as vitamins and minerals. Lactoferrin has anticancer, antiviral, and antibacterial effects, whereas angiogenin promotes blood vessel development, an important factor in cardiovascular health (Musaev et al., 2021). Lactic acid bacteria (LAB) dominate the microflora of fermented milk products, converting lactose to lactic acid. Beyond lactose metabolism, LAB contribute to the nutritional and functional properties of fermented milk by producing bioactive peptides and exerting probiotic effects that enhance gut and immune health. Probiotic strains in fermented dairy products exert competitive inhibition against enteric pathogens that have entered the system. Their most important effect is depriving pathogens of essential resources and adhesion sites. Targeted antimicrobial secretions derived from fermentation further enhance pathogen suppression, and LAB produce various bacteriocins (nisin, plantaricins, reuterin, and pediocins) with strain-specific spectra (Eker et al., 2024; Gao et al., 2025).

There are numerous studies demonstrating the antiviral effects of lactoferrin against various viruses, including SARS-CoV-2, influenza, Zika, and hepatitis C. (Eker et al., 2024). The in vitro efficacy of lactoferrin against viral infections, including SARS-CoV-2, has been demonstrated in several experimental studies, particularly using Vero E6 cells (El-Fakharany et al., 2023; He et al., 2023; Lai et al., 2022; Tufan et al., 2022). Although limited, in vivo studies have investigated the effects of lactoferrin on SARS-CoV-2. However, these studies do not involve challenge trials with live viruses (Tufan et al., 2022). He et al. (2023) investigated the antiviral activity of bovine lactoferrin in 8- to 9-week-old golden hamsters infected with GX_P2V and reported significant results (He et al., 2023). In another study, El-Fakharany et al. (2023) demonstrated that lactoferrin-coated zinc nanoparticles significantly reduced bleomycin-induced lung fibrosis in albino rats (El-Fakharany et al., 2023). In vivo studies have investigated the protective effect of bovine lactoferrin against SARS-CoV-2. While some of these studies did not include challenge experiments with the live virus, the study conducted on golden Syrian hamsters examined only the protective mechanism mediated by bovine lactoferrin, and its findings are consistent with and supportive of our results. Overall, the antiviral efficacy of the traditional fermented drink kumis has not been specifically investigated. Improvements were evidenced by decreases in congestion score, fibrosis, alveolar wall thickness, and inflammation. Additionally, several studies have investigated the antiviral and immunomodulatory potential of lactoferrin in the context of viral infections, including COVID-19. For example, Campione et al. (2021) reported that oral and intranasal administration of liposomal bovine lactoferrin in asymptomatic and mild-to-moderate COVID-19 patients was associated with earlier viral RNA clearance and significant reductions in inflammatory markers such as IL-6, ferritin, and D-dimer levels. In parallel, in vitro studies have demonstrated that liposomal lactoferrin exerts enhanced antiviral activity against SARS-CoV-2 pseudoviruses and other human coronaviruses compared to its free form (Andreu et al., 2023). Furthermore, lactoferrin supplementation has been shown to reduce the incidence and duration of respiratory symptoms in pediatric populations, supporting its broader immunomodulatory role (Motoki et al., 2020). It has been demonstrated by many researchers that the fermented beverage kumis has shown positive effects in the treatment of diseases related to the nervous and digestive systems, respiratory tract, as well as tuberculosis, dysentery, typhoid, paratyphoid, ulcers, and hepatitis. In Russia, both national and international studies have reported that kumis is widely used in the treatment of tuberculosis, particularly pulmonary tuberculosis, and is considered one of the most effective therapeutic agents (Erdem & Gundogdu, 2018).

Antituberculosis drugs used in the treatment of respiratory tuberculosis often lead to colonic dysbacteriosis over time. In one study, cow’s kumis was administered in addition to standard antituberculosis therapy to correct this dysbacteriosis. Among the patients who received kumis, signs of intoxication disappeared in 12% of cases, whereas in the control group, the level of intoxication was observed to double. Similarly, the rate of lung lesion recovery was 62% in the kumis*-*treated group, compared to only 23% in the group that did not consume kumis. The improvement rate of dysbacteriosis was also found to be significantly higher in the kumis-treated patients than in those who did not receive kumis (Dugina et al., 2004).

Ya et al. (2008) conducted an in vivo experimental study to evaluate the immune response in animals using *Lactobacillus casei* Zhang (LcZhang). Oral administration of live LcZhang to BALB/c mice resulted in increased production of interferon-γ (IFN-γ) and decreased tumor necrosis factor-α (TNF-α) levels. Additionally, interleukin-2 (IL-2) and IL-2 receptor gene transcription were significantly upregulated. The study also demonstrated enhanced secretion of intestinal secretory IgA (sIgA), indicating stimulation of gut mucosal immunity. These findings suggest that probiotic strains derived from fermented products such as koumiss may influence both mucosal and systemic immune responses (Ya et al., 2008). Kumis has also been shown to possess antiviral properties in experimental models. In their review, Xue et al. (2023) summarized various studies investigating microorganisms isolated from kumis and their immunomodulatory effects observed in both in vitro and in vivo models. In one of the cited studies, administration of *Lactobacillus casei* Zhang to BALB/c mice was associated with increased IL-2 and total IgG levels, suggesting enhanced T and B cell activation. In another study, *Lactobacillus* and *Lactococcus* strains isolated from kumis were reported to stimulate the production of cytokines such as IFN-β, IL-12, and IL-10 in macrophages and dendritic cells, indicating possible modulation of antiviral immune responses. Overall, previous literature suggests that these microbial strains may be associated with antiviral effects in experimental settings. Additionally, Rizwan et al. (2023) discussed that bioactive peptides derived from fermented foods may exhibit angiotensin-converting enzyme (ACE) inhibitory activity. Through modulation of ACE activity, such peptides could theoretically influence the renin–angiotensin system (RAS) and the conversion of angiotensin I to angiotensin II.

Clinical studies involving kumis rehabilitation in individuals with post-COVID-19 lung complications are among the most significant efforts directly examining the potential benefits of kumis against COVID-19. Gilmutdinova et al. (2021) reported that the addition of kumis therapy to the treatment regimen of 64 patients with COVID-19-related pneumonia significantly accelerated physical recovery (Gilmutdinova et al., 2021). Maikanov et al. (2023) conducted a study in Kazakhstan on the use of kumis-based functional foods during the COVID-19 recovery period (Maikanov et al., 2023). The researchers detailed the development and nutritional properties of a beverage called “balqymyz,” which is produced by fermenting kumis with honey. Due to its high probiotic content and caloric value, this novel beverage is recommended as a supportive dietary supplement in the rehabilitation of patients with lung conditions such as tuberculosis, bronchopneumonia, or COVID-19.

In the study by Istanbullugil et al. (2024), the antibacterial activity of kumis incubated at room temperature (20–25 °C) for different durations was tested using starter cultures. In our study, however, we aimed to evaluate, for the first time, the potential protective effect of kumis against live SARS-CoV-2 in an in vivo model. In this study, mice treated with kumis samples fermented in different containers exhibited significantly less weight loss and lower mortality rates compared to the control group. Between days 12 and 15, control mice lost 22–33% of their body weight and died, whereas mice in the kumis-treated groups experienced milder symptoms. These findings indicate attenuation of specific disease parameters in the experimental mouse model, although the underlying mechanisms remain to be clarified. In the study by Istanbullugil et al. (2025), metagenomic analysis was performed using the same kumis samples fermented in the same types of containers as those used in our study. The results revealed that the microbial community of kumis was predominantly composed of rich LAB species such as *Lactobacillus kefiranofaciens*, *Lactococcus raffinolactis*, *Lactiplantibacillus plantarum*, and *Lactococcus cremoris*. In addition to LAB bacteria, other bacterial species including *Kluyvera intermedia*, *Raoultella planticola*, and *Hafnia alvei* were also isolated and identified as part of the kumis microbiota. The material type of the fermentation container did not significantly affect microbial diversity; however, higher levels of *Bifidobacterium spp*. and bacteriophages were detected in kumis samples fermented in plastic containers. Furthermore, several antimicrobial resistance genes and gene clusters responsible for the production of bioactive compounds were identified in the kumis samples analyzed (Istanbullugil et al., 2025). This distinct microbial composition may contribute to the observed differences between fermentation containers. In particular, Bifidobacterium spp. are known to exert immunomodulatory effects, including the enhancement of mucosal immune responses and the regulation of inflammatory pathways. Additionally, their metabolic activity leads to the production of short-chain fatty acids, which play a role in maintaining epithelial barrier integrity and modulating host immunity. These mechanisms may partly explain the more favorable trends observed in the plastic-fermented kumis group in terms of viral load and histopathological outcomes. In the present study, survival curves, lung pathology scores, and viral load data showed numerically favorable patterns in kumis-treated groups compared to controls; however, these differences did not consistently reach statistical significance. The relatively small sample size (*n* = 6 per group) may have limited the statistical power to detect differences between groups. Nevertheless, effect size estimates suggested moderate effects for several parameters, indicating potential biological relevance despite the lack of statistical significance. Therefore, these findings should be interpreted cautiously and are best considered as preliminary observations suggesting a possible antiviral trend of kumis rather than definitive evidence of protection. Furthermore, our findings indicate that the type of container used for fermenting kumis may influence the observed antiviral-related outcomes. Although numerically favorable effects were noted across treatment groups, occasional mortality in the leather- and wooden-fermented groups suggests that the fermentation environment could potentially contribute to differences in microbial composition and product characteristics. This observation also highlights the importance of fermentation conditions in shaping the biological properties of traditional fermented products. Further studies with larger sample sizes and standardized production conditions are needed to clarify these observations.

Taken together, kumis may have potential as a supportive nutritional intervention during viral infections; however, it should not be regarded as a standalone treatment for COVID-19. As a traditionally produced fermented product, kumis inherently carries microbiological variability, which must be carefully considered in future translational and clinical studies. Comprehensive safety evaluation, standardization, and controlled production will be essential before any adjunct application in humans can be recommended.

## Conclusions

This study provides the first in vivo evidence that kumis, a traditional fermented mare’s milk beverage, can mitigate the severity of SARS-CoV-2 infection in K18-hACE2 transgenic mice. Oral administration of kumis reduced weight loss, lowered viral load and lung pathology, with the strongest effects observed in samples fermented in plastic containers. These findings provide preliminary experimental evidence supporting further investigation of kumis as a potential adjunct nutritional approach.

The strengths of the study include the use of a well-established transgenic mouse model, direct evaluation against live virus under biosafety level 3 conditions, and the comparison of different fermentation containers, which provided insight into the impact of production methods on bioactivity. However, the limitations of the study should also be acknowledged: the small sample size and the absence of mechanistic and long-term data (such as in vitro neutralization assays and immune profiling analyses including cytokine measurements) limit the generalizability of the findings. Taken together, these results suggest that kumis may serve as a promising supportive food during viral infections; however, further research is needed to clarify its mechanisms of action and to confirm its efficacy and safety in humans.

## Supplementary Information

Below is the link to the electronic supplementary material.

Supplementary Material 1

## Data Availability

All data generated or analyzed during this study are included in this published article.
