# Supplementary material for: In vivo Assessment of the Potential Protective Effects of Traditional Kumis Against SARS-CoV-2 in a Transgenic Mouse Model
Source: Food Environ Virol. 2026 Apr 20;18(2):18. doi: 10.1007/s12560-026-09692-4 (PMC13095981; doi:10.1007/s12560-026-09692-4)
Supplement: Supplementary file 1 — Supplementary Material 1 [file 12560_2026_9692_MOESM1_ESM.docx]

| **Dose-Ranging Studies** | **Groups** | **Animal No** | **1. Day** | **7. Day** | **Weight change (%)** |
| --- | --- | --- | --- | --- | --- |
| **Study 1** | **Group1: Plastic** | **1** | 21,74 | 22,22 | 2,16% |
|  |  | **2** | 22,46 | 21,86 | -2,74% |
|  |  | 3 | 22,24 | 22,92 | 2,97% |
|  |  | **4** | 23,5 | 23,95 | 1,88% |
|  | **Group2: Wooden** | **1** | 23,1 | 23,5 | 1,70% |
|  |  | **2** | 22,54 | 22,06 | -2,18% |
|  |  | **3** | 23,04 | 22,53 | -2,26% |
|  |  | **4** | 21,98 | 23,5 | 6,47% |
|  | **Group3: Leather** | **1** | 23,44 | 23,3 | -0,60% |
|  |  | **2** | 22,48 | 23,09 | 2,64% |
|  |  | **3** | 22,38 | 22,63 | 1,10% |
|  |  | **4** | 22,42 | 21,72 | -3,22% |
|  | **Group4: Control (PBS)** | **1** | 23,33 | 24,54 | 4,93% |
|  |  | **2** | 21,98 | 23 | 4,43% |
|  |  | **3** | 22,52 | 22,35 | -0,76% |
|  |  | **4** | 23,41 | 22,7 | -3,13% |

**Table 1.** Body weight changes during acute oral toxicity evaluation of kumis

**Table 2.** In vivo effects of kumis produced in three different fermentation containers on body weight loss, lung gross pathology scores, histopathological scores, and viral RNA (PCR) responses

| **Dose-Ranging Studies** | **Groups** | **Animal No** | **Weight Change (%)** | **Lung Gross Pathology Scores** | **Histopathological Scores** | **Pulmonar Tissue SARS-CoV-2 Real Time PCR Ct** | |
| --- | --- | --- | --- | --- | --- | --- | --- |
|  |  |  |  |  |  | **N1** | **N2** |
| **Challenge Study** | **Group1: Plastic** | **1** | -4,99% | 0 | 1,5 | 40 | 40 |
|  |  | **2** | 3,55% | 0 | 0,5 | 40 | 40 |
|  |  | 3 | -31,10% | 3 | 2 | Ex | Ex |
|  |  | **4** | 0,91% | 0 | 0,5 | 40 | 40 |
|  |  | **5** | -4,64% | 0 | 0,5 | 40 | 40 |
|  |  | **6** | -1,49% | 0 | 1 | 40 | 40 |
|  | **Group2: Wooden** | **1** | -0,87% | 0 | 0,5 | 37 | 40 |
|  |  | **2** | 0,38% | 0 | 0,5 | 39 | 40 |
|  |  | **3** | -17,44% | 3 | 3 | Ex | Ex |
|  |  | **4** | 2,81% | 0 | 1 | 40 | 40 |
|  |  | **5** | 0,00% | 2 | 1,5 | 40 | 40 |
|  |  | **6** | -9,20% | 3 | 3 | Ex | Ex |
|  | **Group3: Leather** | **1** | 1,10% | 0 | 0,5 | 37 | 37 |
|  |  | **2** | -20,13% | 3 | 1,5 | 17 | 17 |
|  |  | **3** | 1,09% | 0 | 0 | 40 | 40 |
|  |  | **4** | -26,25% | 3 | 3 | Ex | Ex |
|  |  | **5** | 4,19% | 0 | 1,5 | 40 | 40 |
|  |  | **6** | 2,40% | 0 | 0 | 40 | 40 |
|  | **Group4: Control (PBS)** | **1** | -33,10% | 2,5 | 3 | Ex | Ex |
|  |  | **2** | -9,32% | 1 | 3 | Ex | Ex |
|  |  | **3** | -29,13% | 3 | 1,5 | 17 | 16 |
|  |  | **4** | -28,37% | 2 | 3 | Ex | Ex |
|  |  | **5** | -22,22% | 2,5 | 3 | 17 | 17 |
|  |  | **6** | -38,89% | 3 | 1,5 | 27 | 26 |
